# Forensic soil provenancing in an urban/suburban setting: A simultaneous multivariate approach

**DOI:** 10.1111/1556-4029.14967

**Published:** 2022-01-17

**Authors:** Patrice de Caritat, Brenda Woods, Timothy Simpson, Christopher Nichols, Lissy Hoogenboom, Adriana Ilheo, Michael G. Aberle, Jurian Hoogewerff

**Affiliations:** ^1^ Australian Federal Police Canberra Australia; ^2^ Geoscience Australia Canberra Australia; ^3^ National Centre for Forensic Studies University of Canberra Bruce Australia; ^4^ UQ Centre for Natural Gas The University of Queensland St Lucia Australia; ^5^ Present address: Australian Federal Police Canberra Australia

**Keywords:** geochemical mapping, geographic information system (GIS), performance analysis, soil forensics, soil properties, Spearman's correlation coefficients (*r_S_*)

## Abstract

Soil is a ubiquitous material at the Earth's surface with potential to be a useful evidence class in forensic and intelligence applications. Compositional data from a soil survey over North Canberra, Australian Capital Territory, are used to develop and test an empirical soil provenancing method. Mineralogical data from Fourier Transform InfraRed spectroscopy (FTIR) and geochemical data from X‐Ray Fluorescence (XRF; for total major oxides) and Inductively Coupled Plasma‐Mass Spectrometry (ICP‐MS; for both total and *aqua regia*‐soluble trace elements) are obtained from the survey's 268 topsoil samples (0–5 cm depth; 1 sample per km^2^). The simultaneous provenancing approach is underpinned by (i) the calculation of Spearman's correlation coefficients (*r_S_*) between an evidentiary sample and all the samples in the database for all variables generated by each analytical method; and (ii) the preparation of an interpolated raster grid of *r_S_* for each evidentiary sample and method resulting in a series of provenance rasters (“heat maps”). The simultaneous provenancing method is tested on the North Canberra soil survey with three “blind” samples representing simulated evidentiary samples. Performance metrics of precision and accuracy indicate that the FTIR (mineralogy) and XRF (geochemistry) analytical methods offer the most precise and accurate provenance predictions. Maximizing the number of analytes/analytical techniques is advantageous in soil provenancing. Despite acknowledged limitations, it is concluded that the empirical soil provenancing approach can play an important role in forensic and intelligence applications.

**Highlights**

- A novel multivariate soil compositional data analytic workflow is proposed for provenancing.
- All variables from an analytical method assessed together for correlation with evidentiary samples.
- Approach can be applied to new or existing soil geochemical/mineralogical survey data.
- Best performing methods found to be FTIR and XRF analysis, followed by Total ICP‐MS.

## 1 INTRODUCTION

This article is a companion to the previously published article entitled “Forensic soil provenancing in an urban/suburban setting: a sequential multivariate approach” by the same authors [1]. Both articles use the same dataset, namely, a soil geochemical survey of North Canberra, Australian Capital Territory (ACT), Australia, to develop and test provenancing techniques. While the previous article demonstrated how soil property maps can be used *sequentially* to match an evidentiary (questioned) sample, the present contribution develops a *simultaneous* approach that considers the multivariable fit of the evidentiary sample against the samples in the survey.

Consequently, for the sake of parsimony, the reader is referred to the open‐access publication by Caritat et al. [1] for details about the context, background, and methods behind the geochemical survey, and this article will focus only on the new data analytics approach.

The aims of the present contribution accordingly are to:

‐ develop the simultaneous multivariate provenancing approach
‐ present results for this method
‐ quantify the performance of this approach
‐ compare the previous sequential method to the simultaneous one
‐ draw conclusions as to the suitability of the simultaneous multivariate provenancing approach to forensic and intelligence applications

## 2 MATERIALS AND METHODS

### 2.1 The North Canberra soil geochemical survey

**FIGURE 1:**
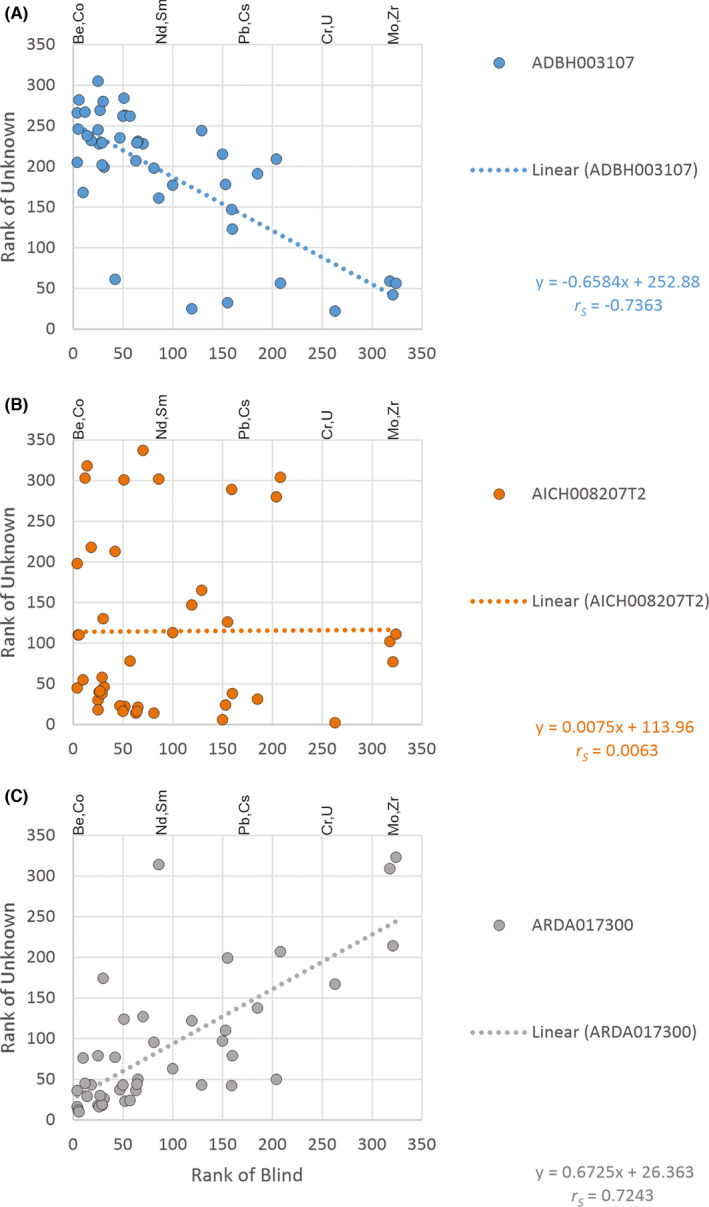
Degree of geochemical similarity (DOGS) scatterplots and linear regressions for Total ICP‐MS trace element ranks in samples ADBH003107 (A), AICH008207T2 (B), and ARDA017300 (C) versus Blind3, showing strongly negative, neutral, and strongly positive Spearman's correlation coefficients (r_S_), respectively. For all plots, selected element ranks in Blind 3 are labeled. Note that *p* ≤ 0.05 for |r_S_| ≥ 0.10 here

A soil geochemical survey of North Canberra initiated in 2017 (see Figure 1 in the companion article [1]) as described in [1, 2]. In addition to the the survey's 268 primary samples (plus 68 additional quality control samples), three “blind” samples (“Blind 1”, “Blind 2”, and “Blind 3” hereafter) were collected from locations unknown to the lead researcher.

Survey details, results, and geo‐environmental interpretations will be reported elsewhere (e.g. [3]). A brief description of the blind samples is presented in the companion article [1] and in Appendix S1 (Figure S1).

We use the following data types in this article: (i) soil mineralogy obtained from infrared spectroscopy (clay minerals, carbonates, sulfates, etc.); and (ii) soil geochemistry (major oxides and organic matter concentrations and trace element concentrations after two chemical extractions of different strength). Appendix S1 contains relevant details about the collection, preparation, and analysis of samples, as well as further information on data analysis, spatial analysis, and quality control.

### 2.2 Simultaneous multivariate provenancing

The soil provenancing method developed here uses the degree of geochemical similarity (DOGS) introduced by Caritat & Mann [4]. Briefly, this method relies on calculating Spearman's correlation coefficient (*r_S_*) between an evidentiary (blind) sample and all the samples from a geochemical database, here the North Canberra geochemical survey database. This is easily achieved using an open‐access spreadsheet application. Because Spearman's rather than Pearson's correlation coefficients are used, the method adequately deals with compositional data issues, such as closure and skewness, as described in [4]. Figure 1 shows the multivariate scatterplots and least‐squares regressions of an evidentiary sample (Blind 3) against three soil geochemical survey samples, one with a strong antithetic correlation (*r_S_* << 0), one with a neutral correlation (*r_S_* ~0), and one with a strong positive correlation (*r_S_* >> 0) to the evidentiary sample. Note that for the North Canberra study, where N = 268, *p* ≤ 0.05 for |*r_S_* | ≥ 0.10. Once *r_S_* has been calculated for every sample site, a map of *r_S_* values is constructed in a GIS, and an interpolated raster is generated as described hereafter.

### 2.3 Raster generation and clipping

Inverse distance weighting (IDW) was used to generate interpolated (gridded) property rasters for all variables, which were subsequently clipped and analyzed (see Appendix S1). All geographic information system (GIS)–related tasks were implemented using the QGIS open‐access software.

### 2.4 Provenancing methodology

A simultaneous multivariate provenancing approach using an empirical database of soil properties is presented here and consists of the following steps. First, measure and map a number of mineralogical (e.g., FTIR) and geochemical (e.g., XRF and ICP‐MS) soil properties at the sampled sites. Second, calculate the DOGS *via* the *r_S_* values for each analytical method, for example, one for FTIR, another for XRF, etc. Third, interpolate those properties between sampled sites, here performed using IDW (power 3; 12 neighbours; 250 m cells) as detailed in Appendix S1. This results in a map for each analytical method with cells having values ranging from −1 to +1 (with the values most antithetically opposite the evidentiary sample under consideration being the most negative, and those most sympathetically like it being the most positive). These interpolated grids are colored to yield “heat maps” that identify areas most like the evidentiary sample, and thus more likely to contain the potential origin for it. Fourth, statistically analyze the resulting DOGS raster, evaluate performance, and test sensitivity to the interpolation parameters.

The present method is designed to work *in combination with*, not at the exclusion of, other provenancing approaches, with the common aim to exclude regions that are least likely to be the source of the evidentiary sample and focus available resources on those that are most likely.

## 3 RESULTS AND DISCUSSION

The collected data are summarized in the companion article (Table 1 in [1]). Similarly, the lower limits of detection can be found in Appendix S1 of the companion article (Table S1 in [1]).

### 3.1 Validation

The target values for the three blind samples are given in the companion article (Table 2 in [1]). The results of soil provenancing investigations using the simultaneous multivariate approach are discussed below.

**FIGURE 2:**
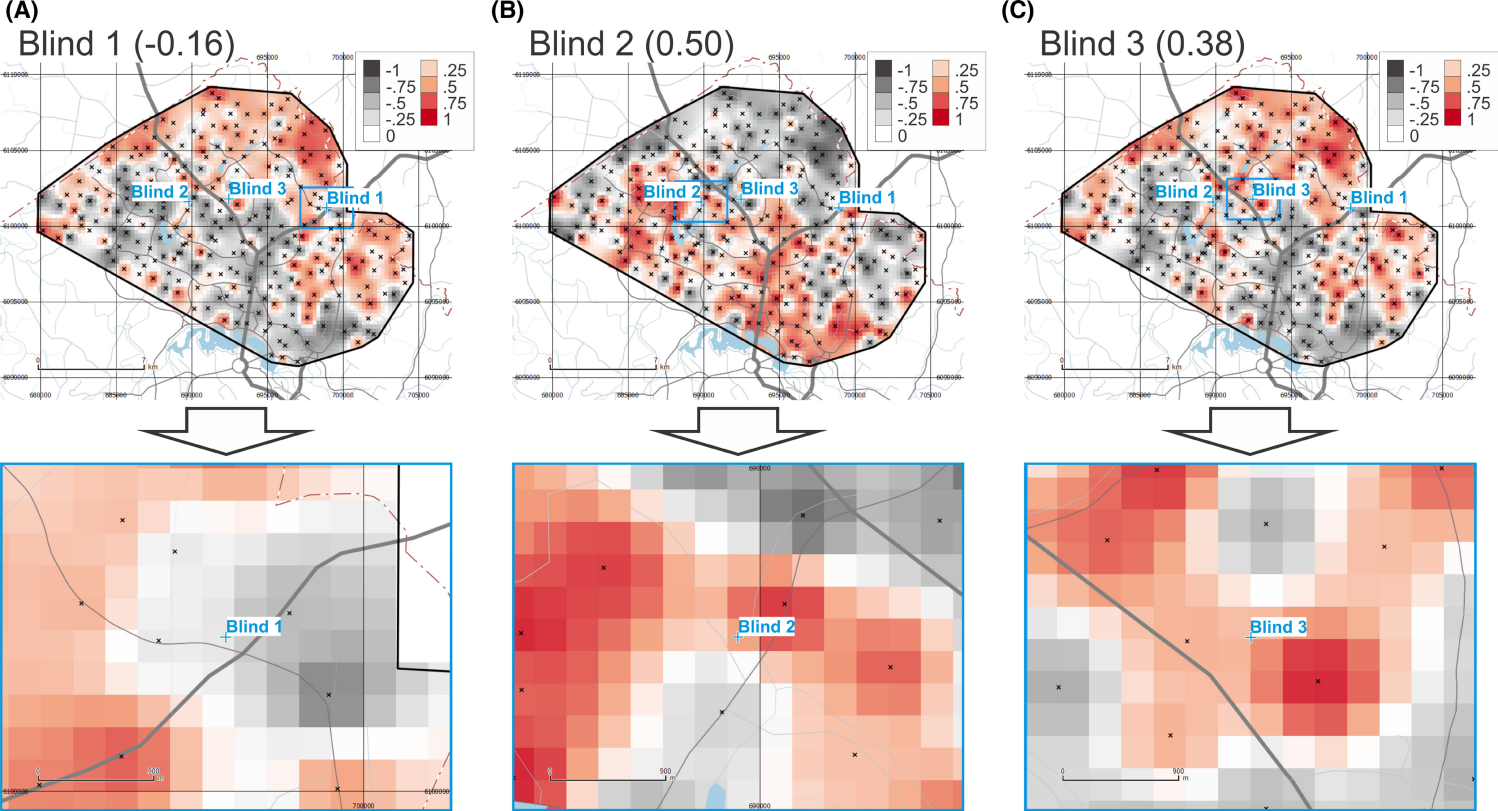
Degree of geochemical similarity (DOGS) provenance prediction maps for unknown samples Blind 1 (A), 2 (B), and 3 (C) for FTIR data (overview on top, detail below). Raster cells are coloured according to Spearman's correlation coefficient (r_S_) on a fixed scale from −1.0 to +1.0. Geospatial data from ACT Government or Australian Government, unless otherwise indicated

The DOGS maps of provenance prediction for samples Blind 1, 2, and 3 based on FTIR data are shown in Figure 2. These maps are based on calculating *r_S_* for 5500 variables (all wavenumbers between 400 and 4000 cm^−1^ after removal of non‐relevant wavelengths between 1800 and 2749 cm^−1^ and normalization of the spectra). Results indicate that for these three blind samples, *r_S_* values of −0.16, 0.50, and 0.38 were obtained for Blind 1, Blind 2, and Blind 3, respectively.

The resulting rasters can be regarded as “heat maps” where grid cells with warmer colors are a better match to the evidentiary sample under investigation than cooler colored cells. In Figure 2A, grid cells colored white or light to dark red (*r_S_* ≥ 0) indicate a match equivalent or superior to the cell from which the simulated evidentiary sample Blind 1 actually comes from (which has an *r_S_* of −0.16). DOGS provenancing grids are all shown with a fixed color scale comprising nine classes of equal range (0.25) between −1 and +1. In the next section (*Performance assessment*), we will discuss metrics to quantify how good the provenance predictions are.

**FIGURE 3:**
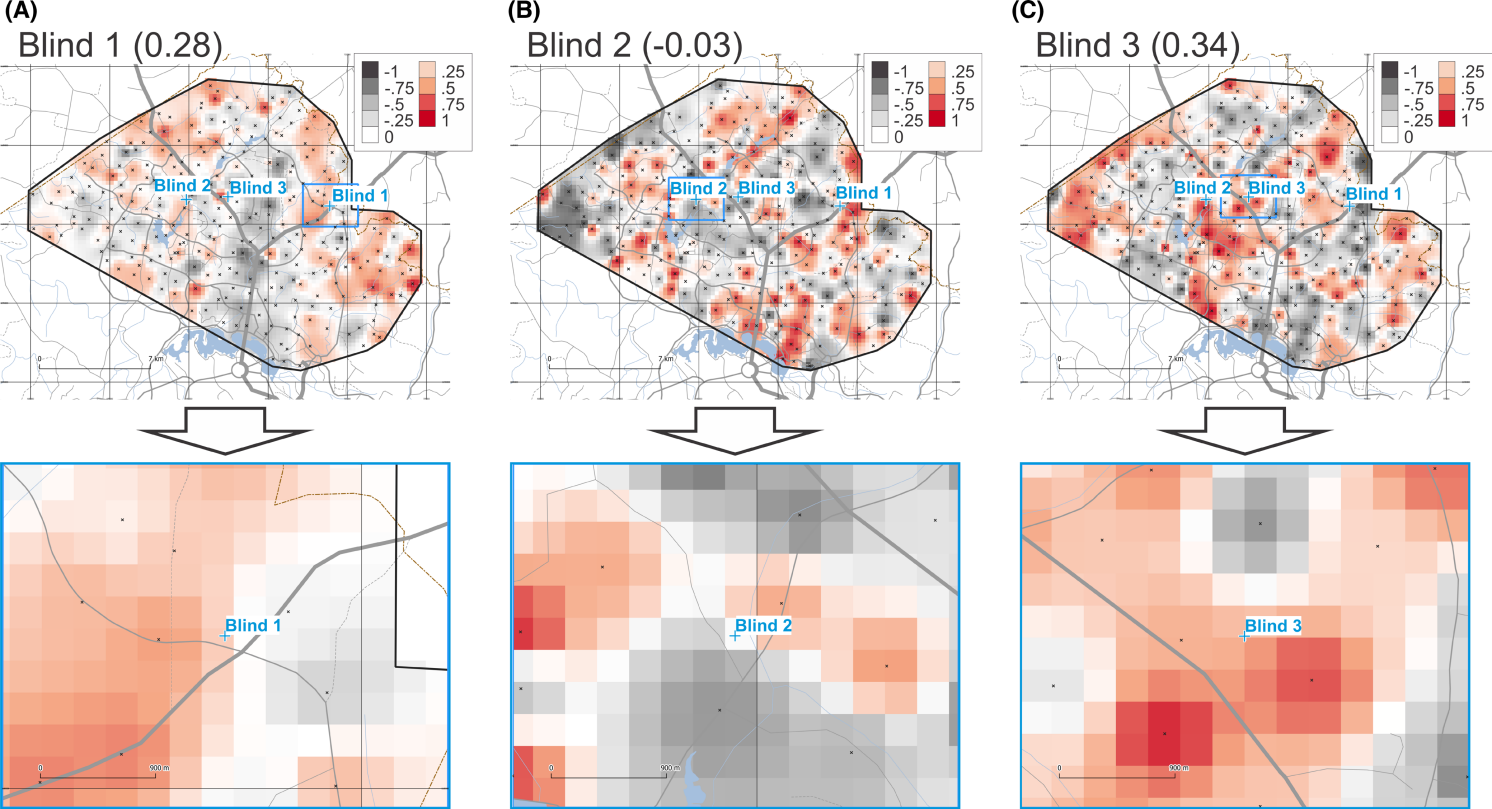
Degree of geochemical similarity (DOGS) provenance prediction maps for unknown samples Blind 1 (A), 2 (B), and 3 (C) for XRF data (overview on top, detail below). Raster cells are coloured according to Spearman's correlation coefficient (r_S_) on a fixed scale from −1.0 to +1.0. See Figure 2 for symbology and credits

The provenancing grids for samples Blind 1, 2, and 3 based on XRF data are shown in Figure 3. These maps are based on calculating *r_S_* for 11 variables (10 major oxide concentrations and LOI). Resulting *r_S_* values of 0.28, −0.03, and 0.34 were obtained for samples Blind 1, 2, and 3, respectively.

**FIGURE 4:**
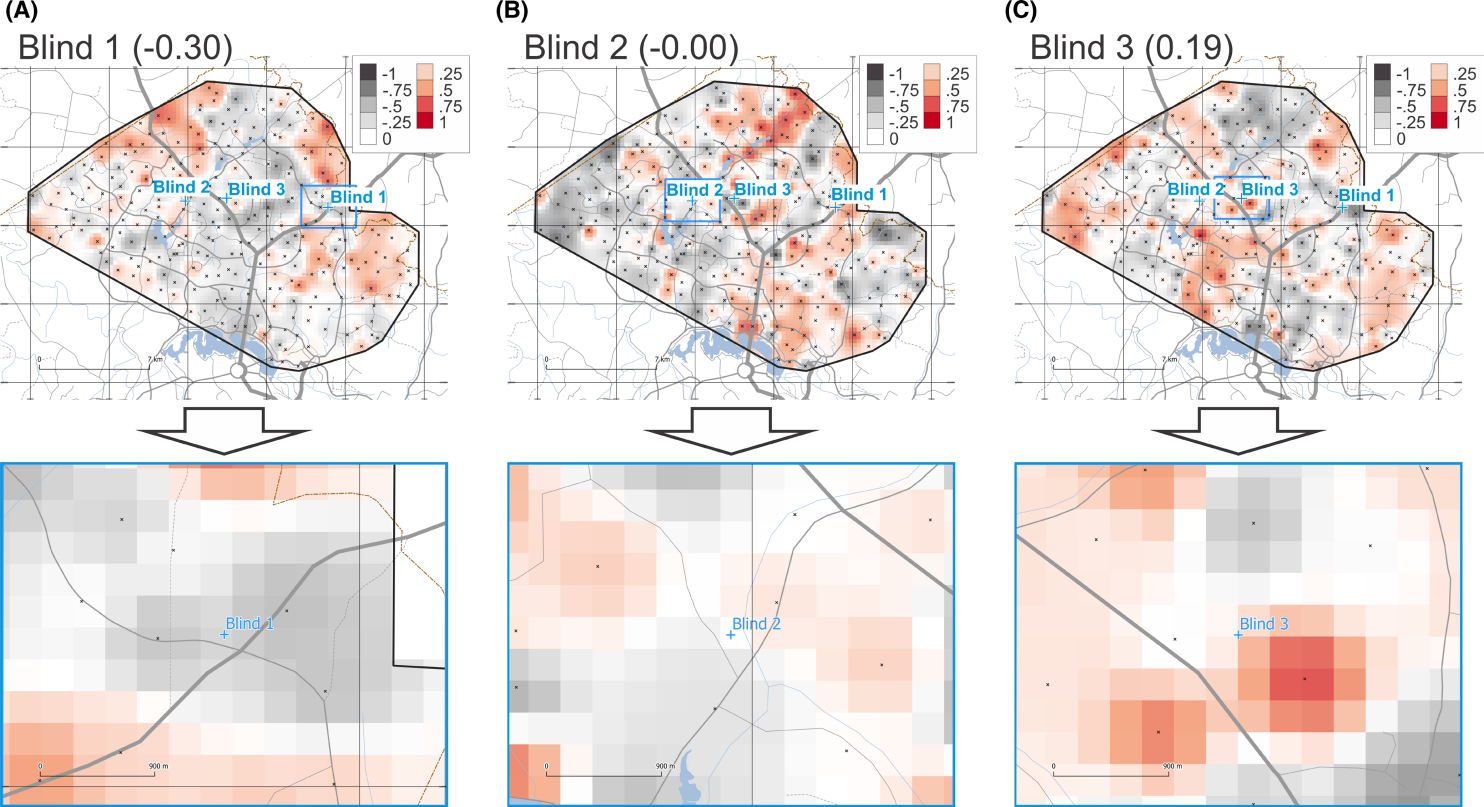
Degree of geochemical similarity (DOGS) provenance prediction maps for unknown samples Blind 1 (A), 2 (B), and 3 (C) for Total ICP‐MS data (overview on top, detail below). Raster cells are coloured according to Spearman's correlation coefficient (r_S_) on a fixed scale from −1.0 to +1.0. See Figure 2 for symbology and credits

The provenancing grids for samples Blind 1, 2, and 3 based on Total ICP‐MS data are shown in Figure 4. These maps are based on calculating *r_S_* for 38 variables (minor and trace element total concentrations). Resulting *r_S_* values of −0.30, −0.00, and 0.19 were obtained for samples Blind 1, 2, and 3, respectively.

**FIGURE 5:**
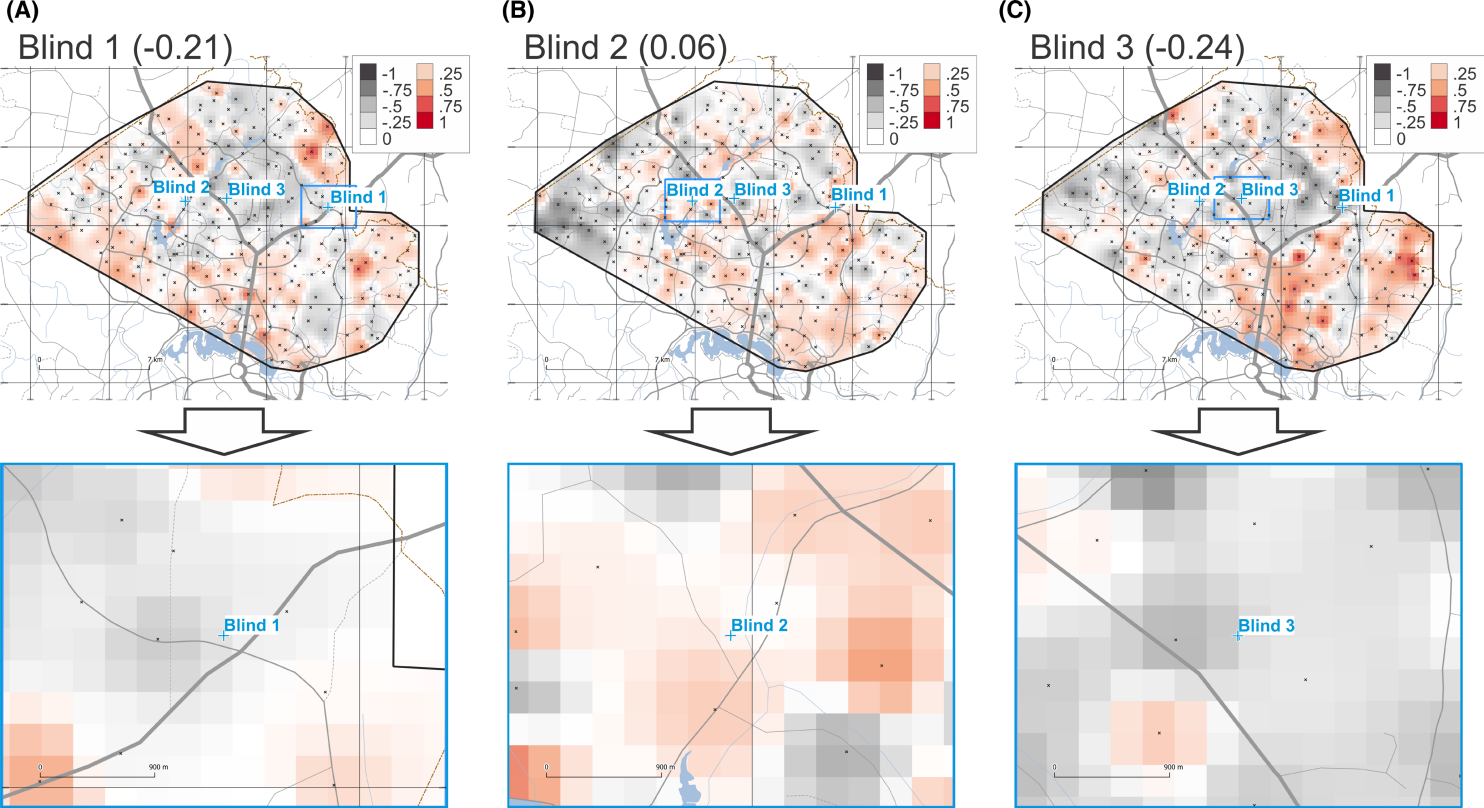
Degree of geochemical similarity (DOGS) provenance prediction maps for unknown samples Blind 1 (A), 2 (B), and 3 (C) for AR ICP‐MS data (overview on top, detail below). Raster cells are coloured according to Spearman's correlation coefficient (r_S_) on a fixed scale from −1.0 to +1.0. See Figure 2 for symbology and credits

The provenancing grids for samples Blind 1, 2, and 3 based on AR ICP‐MS data are shown in Figure 5. These maps are based on calculating *r_S_* for 19 variables (minor and trace element concentrations after *aqua regia* digestion). Resulting *r_S_* values of −0.21, 0.06, and −0.24 were obtained for samples Blind 1, 2, and 3, respectively.

**FIGURE 6:**
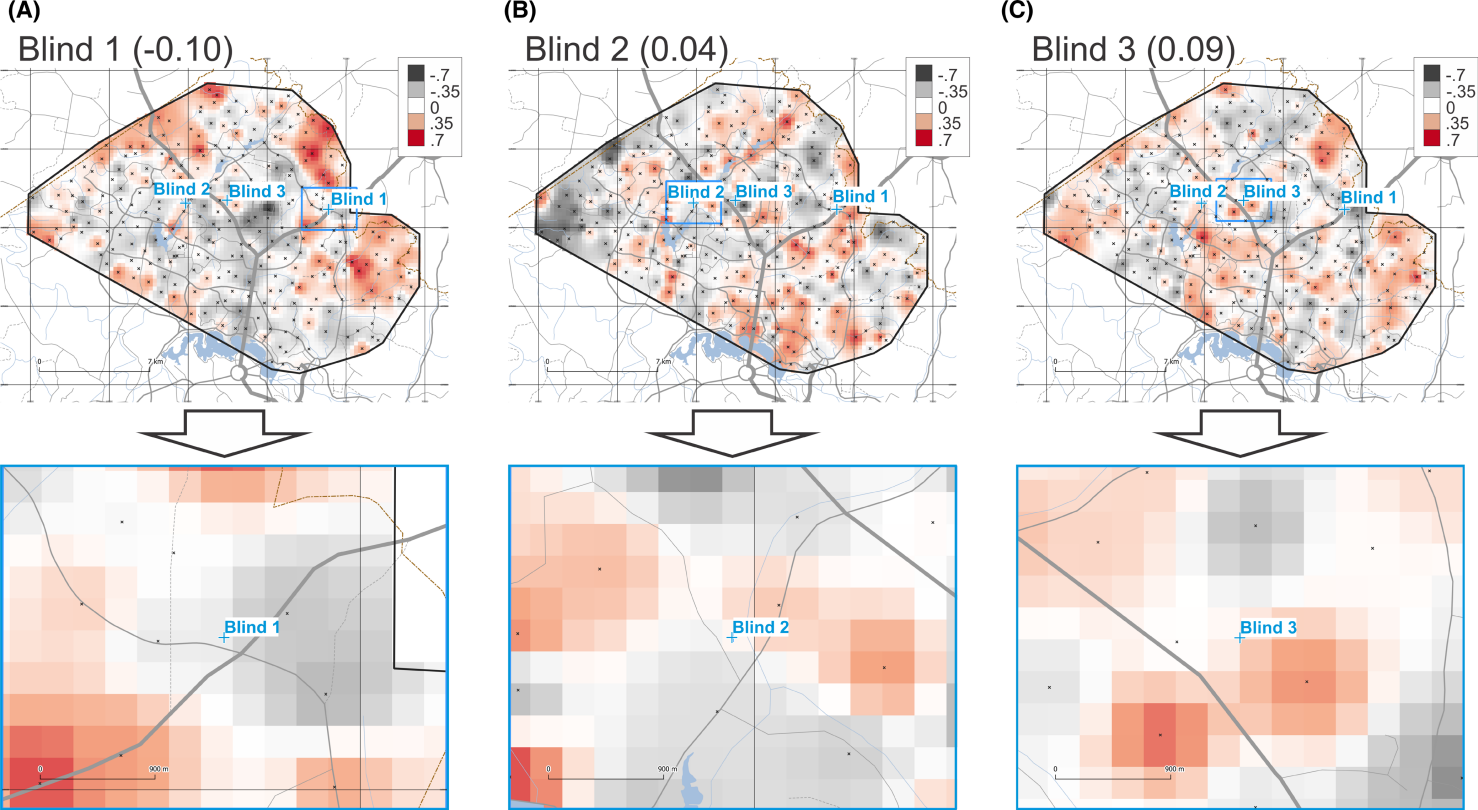
Degree of geochemical similarity (DOGS) provenance prediction maps for unknown samples Blind 1 (A), 2 (B), and 3 (C) for the average values of the FTIR, XRF, Tot ICP‐MS, and AR ICP‐MS DOGS maps. Raster cells are colored according to Spearman's correlation coefficient (r_S_) on a fixed scale from ‐0.7 to +0.7 (5 classes of 0.35 range each). See Figure 2 for credits

The provenancing grids for samples Blind 1, 2, and 3 based on the average *r_S_* from the above‐mentioned four methods (FTIR, XRF, Tot ICP‐MS, and AR ICP‐MS data) are shown in Figure 6. These maps are based on summing the four *r_S_* grids and dividing the resulting values by four (at each pixel). Resulting *r_S_* values of −0.10, 0.04, and 0.09 were obtained for samples Blind 1, 2, and 3, respectively.

### 3.2 Performance assessment

The performance statistics of the simultaneous method of provenancing soil samples are summarized in Table 1. Two performance indicators, precision and accuracy, are defined below.

“Precision” (*Prc*) is the quotient of cells in a grid that have scores equivalent to, or lower than, the score of the cell containing the Blind (evidentiary) sample over the total number of cells. If *Prc* is 90%, only 10% of cells are identified as provenance matches, a highly precise result (effectively excluding 90% of the area). Thus, for instance Blind 1 for FTIR has an *r_S_* of −0.16; there are 1785 cells of the total 4628 cells of the FTIR DOGS grid that have an *r_S_* of −0.16 or less, giving *Prc* = 1785/4628 or 38.6%.

“Accuracy” (*Acc*) is the quotient of the score for the cell containing the Blind (evidentiary) sample in a particular provenancing grid over the (actual) maximum score obtained in any cell within the grid. If *Acc* is 90%, the cell containing the Blind sample belongs to the top 10% of cells in the grid with the highest *r_S_*, a highly accurate result. Thus, for instance, Blind 1 for FTIR has an *r_S_* of −0.16; the grid has a maximum recorded *r_S_* of 0.88, giving *Acc* = −0.16/0.88 or −18.6%. It is noted that because the numerator of *Acc* can be negative, the (theoretical) full range of variation of this performance metric is from −100% to +100%. Negative *Acc* values indicate that soil properties at a particular point in space are more dissimilar than similar to the values interpolated from the soil geochemistry survey points. *Acc* can be rescaled to a positive scale (denoted hereafter by Acc*) of 0%–100% according to:

Acc* = *Acc* + (100 – *Acc*)/2.

**TABLE 1:** Performance statistics for soil provenancing by the simultaneous multivariate method for evidentiary samples Blind 1, 2, and 3 for Fourier Transform InfraRed (FTIR) spectroscopy, X‐ray fluorescence (XRF), and aqua regia (AR) and total (Tot) inductively coupled plasma‐mass spectrometry analytical methods. See text for details

Method	*Prc*	*Acc*
FTIR		
Blind 1	38.6%	−18.6%
Blind 2	79.7%	53.7%
Blind 3	78.0%	44.8%
XRF		
Blind 1	80.2%	31.2%
Blind 2	48.3%	−3.73%
Blind 3	73.0%	34.7%
AR		
Blind 1	17.2%	−27.6%
Blind 2	56.9%	9.79%
Blind 3	20.4%	−31.0%
Tot		
Blind 1	9.62%	−41.2%
Blind 2	53.4%	−0.24%
Blind 3	70.2%	21.5%

Each Blind sample behaves slightly differently in terms of provenancing performance (Table 1). For Blind 1, the method with the highest *Prc* and *Acc* was XRF (80.2% and 31.2%, respectively), followed by FTIR (38.6% and −18.6%, respectively). For Blind 2, the method with the highest *Prc* and *Acc* was FTIR (79.7% and 53.7%, respectively), followed by AR ICP‐MS (56.9% and 9.8%, respectively). For Blind 3, the method with the highest *Prc* and *Acc* was FTIR (*Prc* = 78.0% and *Acc* = 44.8%, respectively), followed by XRF (73.0% and 34.7%, respectively).

Considering *average* (across all methods) instead of maximum and second highest *Prc* and *Acc*, the performance of the simultaneous provenancing approach increased from Blind 1 (36.4% and −14.0%, respectively), to Blind 2 (59.6% and 14.9%, respectively), to Blind 3 (60.4% and 17.5%, respectively). The authors believe that the poorer results for Blind 1 are due to the fact that this sample was collected in a non‐representative location for that grid cell/area (see [1]).

The analytical methods that performed best across all Blind samples were XRF, with the highest *Prc* of any method (80.2% for Blind 1), closely followed by FTIR (79.7% for Blind 2), while FTIR had both the highest and second highest *Acc* of any method (53.7% for Blind 2; 44.8% for Blind 3), followed by XRF (34.7% for Blind 3).

Across all three Blind samples, XRF has the highest average *Prc* (67.2%), closely followed by FTIR (65.4%); FTIR has the highest average *Acc* (26.6%), followed by XRF (20.7%).

### 3.3 Sensitivity analysis

The simultaneous multivariate soil provenancing method developed here consists of a number of steps required for identifying regions within a search area (i.e., cells within a raster) that are more likely to be the source of the evidentiary (blind) sample. In this section, we report on a sensitivity analysis aimed at testing how dependent the results are to parameterization choices. In particular, we measure the effect on the performance metrics *Prc* and *Acc* of (i) using an IDW algorithm with power of 2 (instead of 3) for the interpolation step, (ii) shifting the origin of the interpolation raster grids by 125 m to the west and south; and (iii) using raster grid cells of 500 m × 500 m (instead of 250 m × 250 m). Table 2 shows how these scenarios impact *Prc* and *Acc* for XRF and Total ICP‐MS analyses.

**TABLE 2:** Sensitivity analysis of performance statistics for soil provenancing by the simultaneous multivariate method for evidentiary samples Blind 1, 2, and 3 for X‐ray fluorescence (XRF) and total (Tot) inductively coupled plasma‐mass spectrometry analytical methods. The reference scenario (Sc 0) is compared to three scenarios (Sc 1, 2, and 3) where one interpolation variable is changed each time. See text for details

Method	*Prc*	*Acc*	*Scenario*
XRF			
Blind 1	80.2%	31.2%	Sc 0
	80.2%	27.3%	Sc 1
	62.6%	12.8%	Sc 2
	65.3%	16.7%	Sc 3
Blind 2	48.3%	−3.73%	Sc 0
	36.4%	−15.6%	Sc 1
	17.7%	−45.3%	Sc 2
	39.1%	−15.5%	Sc 3
Blind 3	73.0%	34.7%	Sc 0
	76.8%	33.5%	Sc 1
	79.8%	42.7%	Sc 2
	61.1%	20.2%	Sc 3
Tot			
Blind 1	9.62%	−41.2%	Sc 0
	17.1%	−29.6%	Sc 1
	7.48%	−44.7%	Sc 2
	10.7%	−40.3%	Sc 3
Blind 2	53.4%	−0.24%	Sc 0
	49.3%	−3.87%	Sc 1
	38.0%	−16.7%	Sc 2
	50.0%	−4.41%	Sc 3
Blind 3	70.2%	21.5%	Sc 0
	69.5%	17.8%	Sc 1
	68.5%	20.1%	Sc 2
	49.5%	3.62%	Sc 3

The sensitivity analysis (Table 2) reveals that performance metrics vary mostly within −40% to +10% *relative to the reference scenario* for Blind 1, 2, and 3 combined and that both *Prc* and *Acc* tend to deteriorate (changes relative to the reference scenario ranging between −31% and +8% and between −42% and +12%, respectively) when parameters are altered. Median changes in *Prc* and *Acc* relative to the base scenario are very similar at −3.7% and −4.0%, respectively. The dependency of provenancing performance on parameter choices is relatively significant: performance across all three Blinds and four scenarios averages 50.6% for *Prc* and 0.9% for *Acc*. Therefore, we recommend that values of 50% and 0% be used for *Prc* and *Acc* (or 50% for rescaled Acc*), respectively, as minimum thresholds for accepting a provenance prediction. It is noted that these thresholds still exclude approximately half the survey area from further investigation, resulting in a significant derisking of the provenancing process. On this basis, Table 2 clearly shows that provenancing of Blind 1 was partly successful (8 of 16 performance metrics above the thresholds), provenancing of Blind 2 largely failed (2 of 16), and provenancing of Blind 3 was the most successful (15 of 16).

### 3.4 Comparison to sequential multivariate approach

As the performance metric “Precision” defined above is a direct indication of the efficiency of the provenancing approach, it can be used to compare the present simultaneous multivariate method with the previously published sequential multivariate method [1]. Observing the value of *Prc* indicates what proportion of a provenancing grid can be excluded from further investigation; a higher *Prc* is desirable. The sequential approach [1] resulted in average *Prc* (across all methods and combining with and without principal components, see [1]) of 50.1%, 86.2%, and 70.6% for samples Blind 1, 2, and 3, respectively. In comparison, the simultaneous approach of this contribution resulted in average *Prc* of 36.4%, 59.6%, and 60.4% for samples Blind 1, 2, and 3, respectively.

In terms of “Accuracy” as defined above, the sequential approach [1] resulted in average *Acc* (across all methods and combining with and without principal components, see [1]) of 45.4%, 66.1%, and 46.6% for samples Blind 1, 2, and 3, respectively. In comparison, the simultaneous approach of this contribution resulted in average *Acc* of −14.0%, 14.9%, and 17.5% (or Acc* of 43.0%, 57.4%, and 58.7% when rescaled from 0 to 100% and therefore more directly comparable to the sequential approach[1] numbers) for samples Blind 1, 2, and 3, respectively.

Based on the above, the sequential method [1] is marginally more effective.

When investigating the performance of the two approaches by the method (rather than by evidentiary sample), the sequential approach [1] supported FTIR (and magnetic susceptibility, not included in the simultaneous approach) and XRF as the most precise methods. The simultaneous approach also indicates that FTIR and XRF are the preferred soil analytical methods for provenance analysis.

Finally, the sequential approach requires the preparation of multiple raster grids (one for each variable or soil property), thereby requiring a relatively complex analytic stream, with introduction and management of uncertainty (see [1]). Conversely, the simultaneous approach performs all the similarity calculations at once and only requires the computation of one interpolated grid at the end of the process.

Based on the above, the simultaneous method is simpler and faster to implement, and, therefore, more efficient.

### 3.5 Limitations and future research

This article specifically investigates a data analytic workflow for the provenancing of soil trace evidence under ideal conditions. We acknowledge that, in practice, forensic investigations often have to content with the issues of (i) limited sample size available for analysis; (ii) soil transfer and persistence; and (iii) the potential role of human activity on soil composition. Other limitations to any provenancing approach, such as contamination, are, of course, an important concern and can be managed by appropriate protocols.

Suggestions for future research could include (i) micro‐analysis techniques that accommodate smaller sample sizes; and (ii) quantitative mineralogical and geochemical assessment of soil transfer and persistence.

Despite the acknowledged limitations and recognition that additional research is recommended, we posit that empirical soil provenancing based on soil mineralogical and geochemical surveys can play an important role in forensic and intelligence applications.

## 4 CONCLUSIONS

The multivariate simultaneous provenancing method consists in calculating Spearman's correlation coefficient (*r_S_*) between an evidentiary (blind) sample and all other samples from a geochemical survey across all the variables generated by a particular analytical method. Once these *r_S_* values are known, they are mapped, and an interpolated soil raster map is prepared for each blind sample. The raster grids are “heat maps” showing the pixels where the variables from the survey best match those of the evidentiary sample.

The evidentiary samples performed variably for the different analytical methods: Blind 1 performed overall the worst; Blind 3 the best. We believe this shows that an evidentiary sample may not always be as representative of its grid cell as a sample taken for geochemical mapping purposes. Not surprisingly, natural soil heterogeneity is a challenge that soil forensic provenancing practitioners have to acknowledge.

The most precise (as defined above) analytical methods for soil provenancing identified in this study are XRF (average *Prc* 67.2% across all three Blind samples), closely followed by FTIR (65.4%), and then Total ICP‐MS (44.4%). The most accurate (as defined above) analytical methods are FTIR (average *Acc* 26.6% across all three Blind samples), XRF (20.7%), and then Total ICP‐MS (−6.7%). Although the previously published sequential method slightly outperforms the sequential approach in terms of precision and accuracy, both agree that FTIR and XRF are the most reliable analytical methods among those tested.

In conclusion, we state that (i) the best analytical methods for empirical soil provenancing are FTIR and XRF analysis, followed by Total ICP‐MS, and last AR ICP‐MS; (ii) combining mineralogy (e.g., FTIR here, but potentially also magnetic susceptibility, X‐ray diffraction, etc.) with geochemistry notably improves the performance of soil provenancing; (iii) obtaining as comprehensive an analytical dataset as possible improves the simultaneous multivariate approach; and (iv) although slightly less effective than the sequential approach, the simultaneous approach is simpler to implement and still achieves exclusion of at least 43% of the study area, leading to a material reduction in risk as well as a valuable prioritization of finite financial and human resources.

## Supporting information

Appendix S1
